# July

**Published:** 1890-07-05

**Authors:** 


### JULY.

The season of summer heat is now at hand. Although we never experience any considerable length of heat as compared with, say, the Indian climate, there are yet days when a very fair representation of tropical temperature occurs in this country. The day on which the most heat is received by the earth is, of course, the longest day—supposing the sun to shine from rise to set. But the season of greatest heat is some time after the longest day, and in July. The explanation is that it is at the latter period when the quantity of stored heat is greatest. The daily expenditure of heat has hitherto fallen short of the daily receipts, and so long as this is the case heat is added to the earth. Soon, however, with shortening days, the reverse must occur. But we do not begin to feel it until the month of July has passed. The heat of July is usually mitigated by thunderstorms, when, in the words of Milton, "The blasting vollied thunder rolls." For this reason, July was held by the ancients as sacred to great Jove. The opprobrious term "blasting" is, however, more applicable to the lightning than to the thunder.

July takes its name from Julius Cæsar. It was originally the sixth month of the year, and called *quinctilis.* But on the alteration of the calendar (*vide* article on January) it became the seventh month. Our Saxon ancestors called it "mœd moanth," or mead month, from the meadows being now in full bloom. It was also called "aftera lida," or the latter mild month. July is perhaps not so rich in notable days as some of the other months. On July 3rd the "dog days" begin, and continue till August 11th. It was long an article of faith even among medical men, that dogs go mad more frequently during this hot period than at any other season, and the connection is still generally accepted by the public. But, as a matter of fact and collected statistics, there are not more mad dogs in July than in any other month. Rabies, indeed, does not at all depend upon heat of climate. No dog will suffer from rabies unless bitten by another dog having the disease. The malady is caused by a poison, which must be introduced into the healthy system of animal or man, the result being rabies in the dog, and hydrophobia in the man. Neither rabies nor hydrophobia originate *de novo.* This fact has at length been recognized, leading to the muzzling order now in force, and so offensive to those who do not appreciate the fact that it is quite possible to stamp out rabies altogether if muzzling were thoroughly enforced for a lengthened period. Yet the muzzling order is evaded in every possible manner, and one-half of the dogs we see with so-called muzzles on are not in reality muzzled at all. The apparatus employed permits of the dog opening its mouth to almost the same extent as if nothing were worn. The 4th of July was formerly devoted to St. Martin, the reputed curer of itch. The 15th of July is St. Swithin's day, noted for the popular delusion that if rain falls on this date there will be rain more or less for forty-five succeeding days. Brand tells us that the origin of this belief dates from the year 865, when St. Swithin, Bishop of Winchester, desired to be buried in the open churchyard. But the monks considered it disgraceful for a saint to be laid in the open churchyard, and so resolved to move the body into the choir. It rained so violently on the day fixed, the 15th of July, and for forty-five days afterwards, that the design was abandoned as blasphemous, and instead a chapel was erected over the grave, at which miracles are said to have been performed. July 21st, 1851, is memorable as the date on which the window tax was abolished. Perhaps there never was a tax more indefensible than the window tax. Solar light is essential to health. Absence of solar light is one of the predisposing causes of scurvy. The familiar instance of a potato sprouting in the dark, throwing out blanched shoots towards the light, shows the effect of the absence of solar light on the vegetable world, and there is every reason to believe that insufficiency of solar light exerts a similar deteriorating influence on the animal world. Moreover, a window tax resulted in a smaller number of windows, and a smaller number of windows resulted in a diminished supply of fresh air. But sanitary principles were not much thought of in the early part of the century. Our Chadwick's, and Rawlinsons, and Parkes had not then arisen, and what did for our fathers was supposed to be good enough for us. On July 25th (or St. James' Day old style) oysters were to be eaten, for there was a popular impression that whoever ate oysters on that day would not want money during the remainder of the year. Oysters formerly were not so expensive as they are now. A man recently remarked he wished he had consumed more oysters when they were cheap. But wishes that we had done better in the past are vain and fruitless. Opportunities let slip through our blindness or negligence will not repeat themselves by order of our tardy repentance! July 26th was known as "mace-day," when it was customary to have beans and bacon for dinner—probably a little economy initiated by the old frugal housewives. July 27th is remarkable in the annals of past events for the fall of the Nameu oak in 1813. The Nameu oak was reputed haunted, because, according to Welsh tradition, the body of Howel Sele was immured therein by order of his rival, Owen Glyndur. On July 30th, in 1751, three men were tried at Oxford for the murder of Ruth Osborne by drowning her in a pond because she was reputed to be a witch! The pond wherein this poor creature lost her life was not quite two feet and a-half in depth, "and yet her not sinking was deemed confirmation strong as holy writ, that she was a witch."

